# Human Serum Albumin Protein Corona in Prussian Blue Nanoparticles

**DOI:** 10.3390/nano14161336

**Published:** 2024-08-11

**Authors:** Chiara Colombi, Giacomo Dacarro, Yuri Antonio Diaz Fernandez, Angelo Taglietti, Piersandro Pallavicini, Lavinia Doveri

**Affiliations:** Dipartimento di Chimica, Università degli Studi di Pavia, v. Taramelli, 12, 27100 Pavia, Italy; chiara.colombi04@universitadipavia.it (C.C.); giacomo.dacarro@unipv.it (G.D.); yuriantonio.diazfernandez@unipv.it (Y.A.D.F.); angelo.taglietti@unipv.it (A.T.)

**Keywords:** Prussian blue, nanoparticles, human serum albumin, protein corona, stability

## Abstract

Prussian Blue nanoparticles (PBnps) are now popular in nanomedicine thanks to the FDA approval of PB. Despite the numerous papers suggesting or describing the in vivo use of PBnps, no studies have been carried out on the formation of a protein corona on the PBnp surface and its stabilizing role. In this paper, we studied qualitatively and quantitatively the corona formed by the most abundant protein of blood, human serum albumin (HSA). Cubic PBnps (41 nm side), prepared in citric acid solution at PB concentration 5 × 10^−4^ M, readily form a protein corona by redissolving ultracentrifuged PBnp pellets in HSA solutions, with C_HSA_ ranging from 0.025 to 7.0 mg/mL. The basic decomposition of PBnp@HSA was studied in phosphate buffer at the physiological pH value of 7.4. Increased stability with respect to uncoated PBnps was observed at all concentrations, but a minimum C_HSA_ value of 3.0 mg/mL was determined to obtain stability identical to that observed at serum-like HSA concentrations (35–50 mg/mL). Using a modified Lowry protocol, the quantity of firmly bound HSA in the protein corona (hard corona) was determined for all the C_HSA_ used in the PBnp@HSA synthesis, finding increasing quantities with increasing C_HSA_. In particular, an HSA/PBnp number in the 1500–2300 range was found for C_HSA_ 3.0–7.0 mg/mL, largely exceeding the 180 HSA/PBnp value calculated for an HSA monolayer on a PBnp. Finally, the stabilization brought by the HSA corona allowed us to carry out pH-spectrophotometric titrations on PBnp@HSA in the 3.5-9-0 pH range, revealing a pKa value of 6.68 for the water molecules bound to the Fe^3+^ centers on the PBnp surface, whose deprotonation is responsible for the blue-shift of the PBnp band from 706 nm (acidic solution) to 685 nm (basic solution).

## 1. Introduction

PB is a coordination polymer composed of hexacyanoferrate [Fe^II^(CN)_6_]^4−^ complexes and Fe^3+^ cations. The two give tridimensional infinite ∙∙∙Fe^2+^∙∙∙CN∙∙∙Fe^3+^∙∙∙ sequences, where both Fe^2+^ and Fe^3+^ are octahedral and hexacoordinated [1]. What is commonly called “soluble PB” has the formula KFe^III^[Fe^II^(CN)_6_]∙nH_2_O (n = 1–5), where each Fe^2+^ is coordinated by the C atoms of 6 CN^−^ anions, and each Fe^3+^ is coordinated by 6 N atom of the same anion that belongs to the kinetically inert [Fe^II^(CN)_6_]^4−^ complexes. Soluble PB has a cubic lattice with a face-centered cubic unit cell, with K^+^ cations occupying half of the centers of the cubic cells. Solubility comes from the readiness of this formulation to form small cubic nanocrystals in the 10–200 nm range, yielding colloidal solutions. “Insoluble PB” has instead the formula Fe^III^_4_[Fe^II^(CN)_6_]_3_∙nH_2_O (n = 14–16), in which a quarter of the [Fe^II^(CN)_6_]^4−^ anions are absent and the octahedral coordination around Fe^3+^ is completed by water molecules [2]. Indeed, both “soluble” and “insoluble” PB have the same crystal and molecular structure [3] and have identical properties, including the intense blue color, which is due to a charge transfer (CT) transition in the Fe^2+^∙∙∙CN∙∙∙Fe^3+^ sequence, resulting in an intense band with λ_max_ at 690–710 nm [1]. The factor leading to one or the other form is just the choice of the precursors stoichiometry, i.e., K_4_[Fe(CN)_6_]/FeCl_3_ 1:1 or 3:4 (“soluble” and “insoluble” PB, respectively). The enormous, recent success of PB in the scientific literature [4,5] relies on its easy synthesis as nanoparticles (PBnps), on its biocompatibility, and on the fact that PB has been FDA-approved since in 2003 as Radiogardase^®^, which is an excellent Tl^+^ and Cs^+^ adsorber. However, the FDA-approved Radiogardase^®^ drug is intended for oral administration [6], i.e., for use in an acidic environment (stomach, intestine) [7], where PB is stable. On the contrary, we have recently demonstrated that when the pH is raised to 7 or higher values, including the physiological pH 7.4 (the pH of blood), PBnps quickly degrade [8]. While the NC∙∙∙Fe^2+^ bond in [Fe^II^(CN)_6_]^4-^ is kinetically inert, thanks to the d^6^ low spin nature of the iron cation, the d^5^ high spin Fe^3+^ cation and the CN∙∙∙Fe^3+^ bond are labile. In a neutral/basic environment, the hard Fe^3+^ cation forms hydroxide complexes such as [Fe(OH)_n_]^(3−n)+/−^, and this leads to quick (hours range) disappearance of the characteristic blue color and erosion of the PBnps. In our previous paper, we have shown that a dispersion of uncoated PBnps in the very common DMEM culture medium (that is buffered at pH 7.4 with NaHCO_3_^−^ and NaH_2_PO_4_) undergoes ~100% degradation after 24 h. However, when 10% FBS (fetal bovine serum) is added to DMEM, as typical of in vitro cell treatments, PBnps gain excellent stability, with >95% integrity after 24 h [8]. We hypothesized that this is due to the high concentration of proteins contained in FBS, that quickly form a protective protein corona around PBnps. The same protein corona protective effect, although not mentioned, most probably also holds in the literature papers in which uncoated PBnps were injected in vivo and apparently resisted for >24 h [9,10]. However, despite the many papers claiming the biomedical in vitro and in vivo application of PBnps, no dedicated study was carried out on the formation of the protein corona and its protective effect against PBnp degradation at physiological pH. As we felt that a specific investigation on this topic would be beneficial for the scientific community, due to the increasing number of papers that present or foresee the in vivo application of PBnps, we chose to study the formation of the protein corona formed around PBnps by the most abundant blood protein, human serum albumin (HSA). HSA is the main protein component of blood, with a concentration as high as 35–50 mg/mL [11]. In this paper, we have treated PBnps with a wide range of HSA concentrations (0.025–7.0 mg/mL). Our aim was to determine if there is a minimum HSA concentration sufficient to obtain PBnp@HSA that are both stable against degradation at pH 7.4 and do not aggregate, as this would allow the preparation of PBnp@HSA that could be safely used in in vivo or in vivo-like conditions. Moreover, besides determining the needed HSA concentration in the preparation procedure, we also wanted to determine the HSA quantity actually forming the protein corona in PBnp@HSA in all the synthetic conditions. This has been performed by using the Lowry method for the quantitative determination of proteins in aqueous solution, whose reliability has been tested and established here also in the presence of PBnps. Finally, taking advantage of the HSA protection in the basic pH conditions, the optical properties of PBnps were studied vs. pH, demonstrating for the first time that the water molecules coordinated to the surface Fe^3+^ centers undergo a deprotonation equilibrium that significantly and reversibly modifies the CT band of PB.

## 2. Materials and Methods

### 2.1. Materials

Potassium hexacyanoferrate(II) trihydrate (≥98%), iron(III) chloride hexahydrate (≥97%), citric acid monohydrate(≥98%), sodium hydrogen phosphate dihydrate (≥98%), sodium dihydrogenphosphate dihydrate (≥99%), albumin from human serum lyophilized powder (≥96%), sodium tartrate dihydrate (≥98%), copper sulfate pentahydrate (≥99%), and Folin–Ciocalteu reagent were all purchased by Merck Italia (Milan, Italy).

### 2.2. Synthesis of PBnps

A total of 49.3 mg (0.25 mmol) citric acid monohydrate was dissolved in 50 mL bidistilled water in a flask with 13.5 mg (0.050 mmol) iron(III) chloride hexahydrate. In another flask, 49.3 mg (0.25 mmol) citric acid monohydrate was dissolved in 50 mL bidistilled water with 21.1 mg (0.050 mmol) potassium hexacyanoferrate(II) trihydrate. Both solutions were heated at 60 °C in a thermostated bath. The K_4_[Fe(CN)_6_] solution was poured in 1 min into the FeCl_3_ solution under magnetic stirring. A deep blue solution was immediately obtained that was further stirred for 5 min at 60 °C and then allowed to cool to room temperature. The PBnp colloidal solution was subdivided into ten 10 mL ultracentrifuge test tubes, and ultracentrifugation was carried out for 45 min at 13,000 rpm (15,870× *g*). The supernatant was of a pale blue color and was removed except for a 0.50 mL volume over the pellet (to avoid any accidental removal of part of the latter). A total of 9.5 mL bidistilled water was added, in which the pellet smoothly dissolved. The procedure was repeated one more time to get rid of excess citric acid. The citrate-coated PBnps were indefinitely stable in the final solution, which had a pH of 4.5–5.5.

### 2.3. Synthesis of HSA-Coated PBnps (PBnp@HSA)

HSA solutions were prepared by dissolving the appropriate mass of HSA in 10.0–100.0 mL bidistilled water so as to have concentrations of 0.025, 0.05, 0.1, 0.25, 0.5, 1.0, 3.0, 5.0, and 7.0 mg HSA/mL. Portions of 10 mL of the PBnp solutions prepared as described in Section 2.2 were ultracentrifuged for 45 min at 13,000 rpm (15,870× *g*), the supernatant removed except 0.5 mL over the pellet, and 9.5 mL of an HSA solution at the chosen concentration was added. The PBnp pellet easily redissolved and was allowed to equilibrate at room temperature for 2 h, after which time ultracentrifugation was carried out (45 min at 13,000 rpm, 15,870× *g*). The supernatant, except 0.5 mL over the pellet, was removed, and the pellet redissolved by the addition of 9.5 mL bidistilled water. The PBnp@HSA solutions had a pH in the 5.5–5.9 range. Their absorption band had λ_max_ = 706 nm.

### 2.4. Phosphate Buffer at pH 7.4

The phosphate buffer at pH 7.4 was prepared by dissolving 0.6917 g NaH_2_PO_4_∙2H_2_O (4.43 mmol) and 1.1127 g (6.25 mmol) Na_2_HPO_4_∙2H_2_O in 100 mL and adjusting the pH to 7.4 with microadditions of standard 1.0 M NaOH.

### 2.5. PBnp@HSA Stability in Phosphate Buffer at pH 7.4

Samples of 10 mL of PBnp@HSA solutions obtained as described in 2.3 were diluted with an addition of bidistilled water (2.9–3.0 mL) so to reach an absorbance value of 1.5 at the peak maximum (706 nm). A total of 2.0 mL of these solutions were treated with (a) 1.0 mL of water and (b) 1.0 mL of phosphate buffer. The absorbance value was 1.0 in case (a), with λ_max_ = 710 nm. In case (b), a spectrum was recorded immediately after mixing (t = 0), and the peak shifted in all cases at 685–686 nm, with an absorbance at λ_max_ = 0.8. Spectra were then recorded every hour for 24 h.

### 2.6. PBnp Stability in Serum-like Conditions

A total of 12 mg HSA was dissolved in 100 μL phosphate buffer, prepared as in Section 2.4 (HSA concentration = 120 mg/mL). A freshly prepared 10mL solution of citrate-stabilized PBnps, prepared by mixing K_4_[FeCN)_6_] and FeCl_3_ solutions as described in Section 2.2, was ultracentrifuged one time for 45 min at 13,000 rpm (15,870× *g*). The supernatant was removed except for 0.5 mL, 9.5 mL bidistilled water was added, and the pellet was redissolved. After the second ultracentrifugation cycle and removal of 9.5 mL supernatant, 0.5 mL bidistilled water was added, and the pellet was redissolved. By this, a 10× concentrated PBnp solution was obtained. A total of 200 μL of the 10× PBnp solution was diluted with 100 μL bidistilled water so as to obtain a solution with Abs at λ_max_ = 1.5 (0.1 cm cuvette). A total of 200 μL of this solution was mixed with 100 μL of the HSA solution in phosphate buffer, and a spectrum was immediately recorded. Spectra were then taken every 6 h for 24 h. The concentration of HSA in this solution was 40 mg/mL, mimicking the HSA concentration in blood (35–50 mg/mL [11]).

### 2.7. pH-Spectrophotometric Titration of PBnp@HSA

A 24 mL volume of PBnp@HSA solution prepared as described in Section 2.3 with 1.0 mg/mL HSA was treated in a 50 mL beaker with 100 μL HCl 0.05 M. The pH of the solution dropped to 3.51. A pH-spectrophotometric titration was carried out on this solution by microadditions (20–60 μL) of 0.05 M NaOH. On the starting solution and after each addition, we measured the pH, then a 2.5 mL portion of the solution was transferred to a quartz cuvette, the absorbance spectrum, hydrodynamic radius, and zeta-potential were measured, and the portion was returned to the titration beaker. Titration was interrupted at pH 9.

### 2.8. Quantitative Determination of HSA with Folin Reagent

We modified the Lowry method [12], which uses the Folin–Ciocalteu reagent for the quantitative determination of proteins. This is a colorimetric method that is based on the ability of the peptides of proteins to coordinate Cu^2+^ in water, with the developed color increased by further coordination of the tungstate and molybdate anions contained in the Folin–Ciocalteu reagent. Absorbance at 750 nm is proportional to the total protein content of a solution [12]. However, PB also absorbs in a similar range. Due to this, we first determined a calibration curve using PBnp solutions in water as the solvent (instead of pure water), in which measured quantities of HSA were dissolved and treated following the Lowry method with the Folin–Ciocalteu reagent. Then, for all the solutions of PBnp@HSA prepared with the different C_HSA_ values (see Section 2.3), we separated by ultracentrifugation the pellet containing PBnp@HSA and the supernatant (containing only HSA not adhering to the PBnps). The pellet of PBnp@HSA was redissolved in water. Using the calibration curve, we determined the concentration of HSA in the supernatant (i.e., not bound to PBnps) and in the redissolved pellet (i.e., HSA forming the protein corona). A detailed description of all the steps follows. A critical description of the method and a discussion of the results can be found in Section 3.4.

#### 2.8.1. Calibration Curve

Five stock solutions were prepared. A: 1.4504 g (0.0137 mol) Na_2_CO_3_ dissolved in 50 mL NaOH 0.143 M; B: 0.1452 g (5.82 × 10^−4^ mol) CuSO_4_·5H_2_O dissolved in 10 mL bidistilled water; C: 0.2894 g sodium tartrate dihydrate (Na_2_C_4_H_4_O_6_·2H_2_O, 1.258 × 10^−3^ mol) dissolved in 10 mL bidistilled water. D: solution A (50 mL) + 0.5 mL solution B + 0.5 mL solution C; E: 0.0208 g HSA dissolved in 100 mL of PBnp solution, freshly prepared as described in Section 2.2 (HSA concentration: 208 µg/mL). Eight HSA standard solutions of 5.0 mL volume were then prepared by mixing 0.1, 0.3, 0.5, 1.0, 1.5, 2.0, 2.5, and 3.0 mL of solution E with the complement to 5.0 mL of bidistilled water. A blank solution was also prepared (5.0 mL bidistilled water). A total of 0.70 mL solution D was then added to each of the 8 solutions and to the blank and allowed to equilibrate in the dark for 20 min. After this, to each solution (blank included), we added 0.1 mL of 1 N Folin–Ciocalteu solution (i.e., 1:1 dilution of the commercial reagent), and the nine samples were allowed to equilibrate in the dark for 30 min, after which time they were filtered using a standard 0.40 μM syringe filter and their absorption spectrum measured to obtain a set of Abs_750_ vs. HSA concentration (C_HSA_) data (see SI1 for the series of spectra). Table 1 summarizes the added quantities and the HSA concentration in the examined solutions.

The same standards were prepared three times, so for each C_HSA_ we collected three Abs_750_ values. For C_HSA_ > 60 μg/mL, the three Abs_750_ values for a given C_HSA_ displayed large deviations from their average (see also Results and Discussion) but with barely different average Abs_750_ values and so these points were discarded. For lower C_HSA_ values, the points were fitted with the cubic curve y = ax + bx^2^ + cx^3^ + y_0_ (x = C_HSA_, y = Abs_750_), finding a = 0.0164, b = −2.2894 × 10^−4^, c = 1.3936 × 10^−6^, and y_0_ = −0.0145, with R^2^ = 0.9953.

For the sake of comparison, a calibration curve was also obtained with the same technique and quantities but with solution E made only of HSA in bidistilled water (i.e., without PBnps); see SI2.

#### 2.8.2. Determination of HSA

PBnp@HSA solutions were prepared as described in Section 2.3. In a typical experiment, after equilibration of PBnps with the HSA solution of the chosen concentration (room temperature, 2 h), ultracentrifugation was carried out for 40 min at 13,000 rpm (15,870× *g*), repeated two times. A total of 9.5 mL of the supernatant was removed and used for C_HSA_ determination (s-C_HSA_). A total of 9.5 mL bidistilled water was added to the pellet with the remaining 0.5 mL supernatant and sonicated until complete dissolution. This solution was analyzed to determine the p-C_HSA_, i.e., the HSA concentration bound to PBnps in the pellet. It has to be stressed that the 10 mL redissolved pellet solution contained 0.5 mL of not removed supernatant. Consequently, the p-C_HSA_ values were corrected by subtracting 0.05 × s-C_HSA_. In a typical analysis of the supernatant or pellet solution, 3–8 portions in the 0.1–4 mL volume range were diluted with the complement to 5 mL of bidistilled water and treated with 0.70 mL solution D and 0.10 mL of 1 N Folin reagent. The rationale was to obtain solutions with HSA concentrations < 60 μg/mL (in case of higher found concentrations, the point was discarded, as it was outside of the calibration curve and higher dilutions were prepared). Equilibration in the dark was allowed for 30 min, after which time the solutions were filtered with a standard 0.40 μM syringe and their absorption spectrum was measured. The Abs_750_ value allowed the calculation of C_HSA_ for each sample using the calibration curve determined in Section 2.8.1. The s-C_HSA_ and p-C_HSA_ values were expressed as the average of the values found for the examined samples. For all the HSA concentrations used in Section 2.3, the sample preparation and C_HSA_ analysis were repeated three times. The actual quantity of HSA adsorbed on PBnps (i.e., forming the hard corona in PBnp@HSA) can be calculated by multiplying the determined concentration by the volume of the solution. The quantity of PBnps contained in the volume can also be calculated from the starting reagent quantities used for the synthesis and from the yield (see Section 2.9).

### 2.9. Determination of the PBnp Yield

A total of 0.5 mL of a 10 mL sample of citrate-coated PBnps, prepared as described in Section 2.2, was added to 5.0 mL bidistilled water and treated with 0.100 mL 0.5 M NaOH. The solution became quickly colorless and was allowed to react for 10 min, after which time 0.2 mL of ultrapure concentrated HNO_3_ (70% w/w) was added. The colorless solution was analyzed by ICP-OES to determine the total Fe content. Comparison with the total Fe added in the starting step of the synthesis (see Section 2.2) allowed us to determine the yield. The procedure was repeated 3 times, calculating an average yield of 44(3)%.

### 2.10. Instrumentation

Absorption spectra were taken on a Varian Cary 50 or an Agilent Cary 60 spectrophotometer with glass or quartz cuvettes (1.0 or 0.1 cm optical path). Dynamic Light Scattering determinations were carried out with a Zetasizer Nano ZS90 Malvern instrument, with a dedicated dip cell accessory for the zeta-potential determination. TEM images were taken with a JEOL JEM-1200 EX II 140 (JEOL Italia, Milano, Italy) electronic microscope. ICP-OES analysis was carried out on an Optima 3000 DW system (Perkin Elmer Italia, Milano, Italy).

### 2.11. Statistical Analysis

Statistical analysis of the data (Figures 3C and 4B) was performed using IBM SPSS statistics 20.0 software. The data were ranked, and statistical differences were evaluated using a one-way analysis of variance (ANOVA) and Turkey’s multiple comparison tests. In all cases, a *p*-value < 0.05 was considered significant.

## 3. Results and Discussion

### 3.1. Synthesis of PBnps and Their HSA Coating

**Figure 1:**
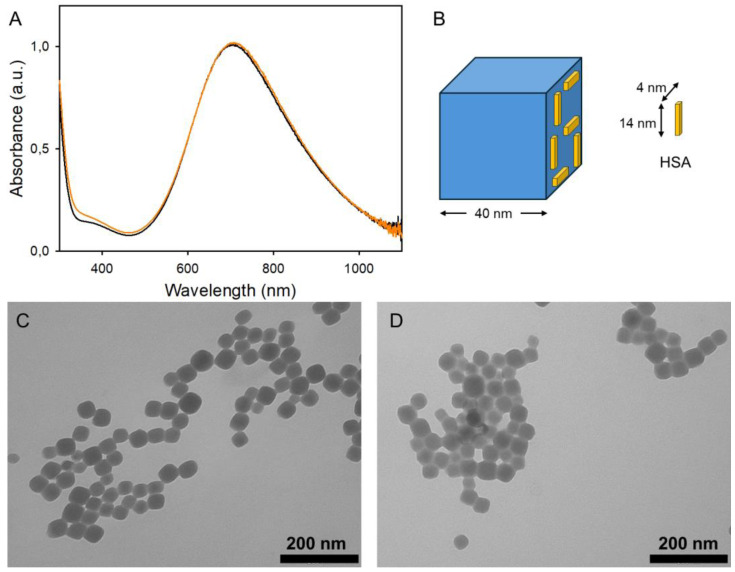
(**A**) Absorption spectrum of citrate-coated PBnps (black) and of PBnp@HSA obtained with C_HSA_ 1 mg/mL (orange); (**B**) PBnp and albumin (modelized as a rectangular object) sketched maintaining the authentic proportions between their dimensions; (**C**) TEM image of citrate PBnps; (**D**) same, coated with C_HSA_ 1 mg/mL.

The PBnps studied in this paper were prepared with the well-established synthetic route in sodium citrate [13]. Equimolar quantities of [Fe(CN)_6_]^4−^ and Fe^3+^ (from K_4_[FeCN)_4_]∙3H_2_O and FeCl_3_∙6H_2_O, respectively), each at a 5 × 10^−4^ M concentration, were mixed in a 5 × 10^−3^ M solution of citric acid in water. The citrate-coated PBnps formed rapidly and were purified with two ultracentrifugation, supernatant discard, pellet redissolution cycles, after which slightly acidic aqueous solutions (pH 5.3–5.4) were obtained. These PBnp solutions are intensely blue colored and indefinitely stable in water, with the characteristic charge transfer absorption band centered at 706 nm (Figure 1A).

The PBnps prepared in this work are cubic, with an average side dimension of 41(8) nm (Figure 1C) (see also SI3 for dimensional distribution). Due to the stoichiometry of the synthesis, their formula is that of “soluble PB”, i.e., KFe^III^[Fe^II^(CN)_6_]∙nH_2_O (n = 1–5). In a previous paper, we demonstrated that citrate-coated PBnps are not stable at physiological pH (7.4) in inorganic buffers and in buffered nutrient culture medium, such as DMEM, unless a 10% fetal bovine serum (FBS) supplement is added, forming a protein corona. In the context of the enormous interest in PBnps for in vivo nanomedical applications, with the perspective of their clinical use on human beings, we decided to study quantitatively the stabilization provided by the protein corona formed by the most abundant protein in human serum, HSA. HSA has a molecular weight (mw) of 66,348 and a concentration in human serum ranging from 35 to 50 mg/mL [11]. Considering the lowest physiological concentration, 35 mg/mL corresponds to 5.27 × 10^−7^ M albumin. Although being subject to deformations depending on the nature of the absorbing surface [14] or to denaturation, in solution, albumin is an ellipsoid with a two-dimensional projection of 14 × 4 nm [15]. For an approximate calculation, we considered HSA as a rectangle with the same dimensions, thus occupying an area of 56 nm^2^. A 41 nm PBnp has a total surface area of 10,086 nm^2^, leading to a number of ~180 HSA molecules needed to form a monolayer on a single PBnp. Figure 1B maintains the proportions of the above-discussed dimensions, providing a visual representation of the space occupied by HSA on PBnps (please note that the third dimension added, i.e., the thickness of the parallelepiped used to represent HSA molecules, is merely indicative, and not based on experimental or literature data). To evaluate the minimum HSA concentration (C_HSA_) needed to fully coat the PBnps prepared in this paper, we used these parameters: the PB density (1.8 g/cm^3^), giving 1.24 × 10^−16^ g mass for a single cubic PBnp with 41 nm side; a PBnp molar concentration of 1.17 × 10^−9^ M, calculated considering the experimentally determined 44(3)% yield after the two ultracentrifugation purification steps, as described in Section 2.2; a KFe^III^[Fe^II^(CN)_6_]∙nH_2_O formula, with n = 5 when dissolved in water (molar mass = 397.001); and the calculated 180 HSA/PBnp for coating a PBnp with an HSA monolayer. This leads to a minimum 2.11 × 10^−7^ M HSA, i.e., C_HSA_ > 0.015 mg/mL.

With this in mind, we prepared PBnp@HSA, i.e., HSA-coated PBnps, by treating pellets of PBnps, as described in Section 2.3, with HSA solutions with C_HSA_ starting from a slight excess with respect to the calculated minimum and progressively increasing it: C_HSA_ = 0.025, 0.05, 0.1, 0.25, 0.5, 1.0, 3.0, 5.0, and 7.0 mg/mL. Equilibration was allowed for 2 h, a sufficiently long time considering that the protein corona formation is a fast phenomenon (minutes range) [16]. Ultracentrifugation and pellet redissolution in bidistilled water followed to get rid of the excess HSA and of the so-called “soft” protein corona [17], producing PBnp@HSA that bear only tightly bound HSA proteins, which are the subject of the studies in this paper.

The hydrodynamic diameter (d_h_) measured for citrate-coated PBnps with DLS experiments in water is 72(1) nm after the standard preparation procedure (2 ultracentrifugation cycles, pH 4–5). DLS displays as expected increased dimensions for all PBnp@HSA (Table 2). However, it must be stressed that d_h_ values are exceedingly large and have a high polydispersity index when PBnps have been treated with low quantities of HSA (finding d_h_ > 200 nm for C_HSA_ ≤ 0.1 mg/mL), while the d_h_ values decrease on increasing C_HSA_, with d_h_ = 123, 112, and 113 nm for C_HSA_ 3.0, 5.0, and 7.0 mg/mL, respectively. This counterintuitive behavior is due to the tendency of PBnps to form random aggregates (resulting also in high PDI) when low quantities of HSA are added. HSA acts as glue, bridging among PBnps, driving to aggregates with d_h_ largely exceeding the d_h_ value of PBnps with no added albumin (72 nm). On the contrary, when larger quantities of HSA are added during the PBnp@HSA preparation, the mass action pushes the system toward single, separate, not aggregating PBnps enveloped in an HSA corona. In PBnp@HSA, the PB core dimensions remain unchanged, as shown in Figure 1D for the case C_HSA_ = 1 mg/mL. A less electron-absorbing HSA halo is clearly visible around the PBnp (see SI3 for additional images). Also, the absorption spectrum of PBnp@HSA remains unchanged with respect to the citrate-coated PBnp (Figure 1A, orange spectrum). The pH of all as-prepared PBnp@HSA solutions is in the 5.5–5.8 range, and no spectral changes are observed after 48 h.

### 3.2. Spectral Changes with pH in PBnp@HSA

**Figure 2:**
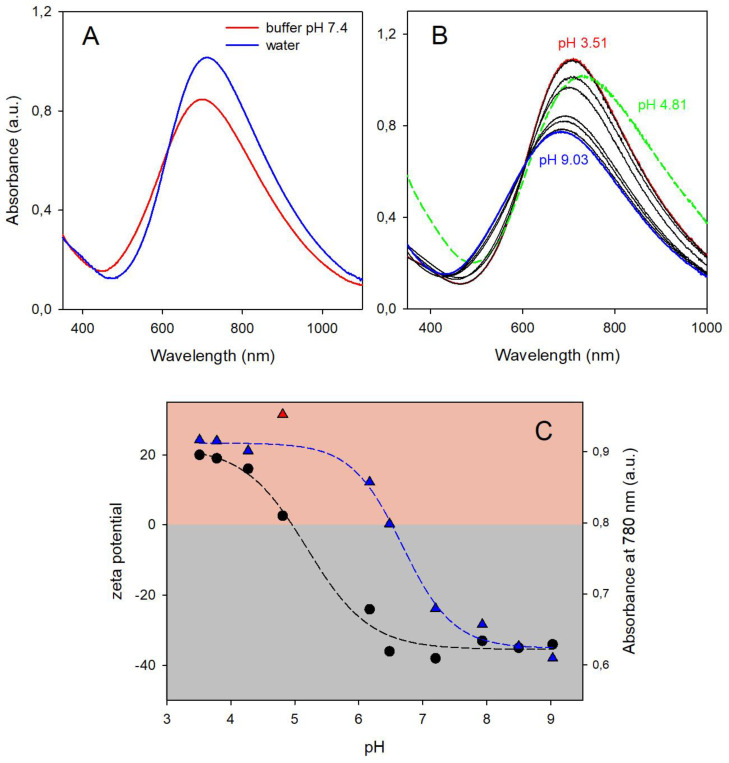
(**A**) Blue spectrum: 2.0 mL PBnp@HSA solution (prepared with C_HSA_ 1 mg/mL) diluted with 1 mL water, pH 5.8; red spectrum, 2.0 mL of the same PBnp@HSA solution, diluted with 1 mL phosphate buffer at pH 7.4; (**B**) absorption spectra recorded during the titration with microadditions of 0.05 M NaOH of an acidified PBnp@HSA solution (C_HSA_ 1 mg/mL); the first spectrum (red, pH 3.51) and the final one (blue, pH 9.03) has been colored to stress the trend of the titration; the spectrum obtained at pH 4.81, affected by scattering due to turbidity, has been colored in green, with dashed line. (**C**) Plots of zeta-potential (black circles) and Abs_780_ (blue triangles) vs. pH; the dashed lines are plots of the sigmoidal fittings of the data (R^2^ = 0.989 and 0.993, respectively), with calculated inflection points at pH 5.18 (zeta-potential) and 6.68 (Abs_780_). The red triangle data (Abs_780_ at pH 4.81) has been excluded from data fitting.

The absorbance of citrate-coated PBnps decreases at basic values in the hours range (e.g., −10% and −19% after 1 and 2 h at pH 7.4 in phosphate buffer [1]). On the other hand, the protection provided by the protein corona significantly slows down the process, as we have observed with FBS [8] and as it is demonstrated here with HSA (see below). This allowed us to use PBnp@HSA to examine the spectral changes vs. pH of PBnp solutions, a phenomenon that has been completely neglected before in the literature and that gives apparently puzzling results. In particular, a remarkable blue-shift with intensity decrease is observed when an acidic solution of PBnps is added to a buffer at pH 7.4. The change takes place in the mixing time (seconds range). Figure 2A compares the spectrum of a PBnp@HSA solution (pH 5.6) diluted either with phosphate buffer at pH 7.4 (red spectrum) or with the same volume of bidistilled water (blue spectrum, pH 5.7). As we pointed out in the introduction, the Fe^3+^ center of “soluble” PB is fully coordinated by the N atoms of the cyanide anions. However, the Fe^3+^ centers on the surface of a PBnp have five coordination positions occupied by the N atom of cyanide anions and one occupied by water. Moreover, the number of Fe^3+^ coordination positions occupied by H_2_O is >1 on the edges, vertices, and defects of a PBnp.

**Figure 3:**
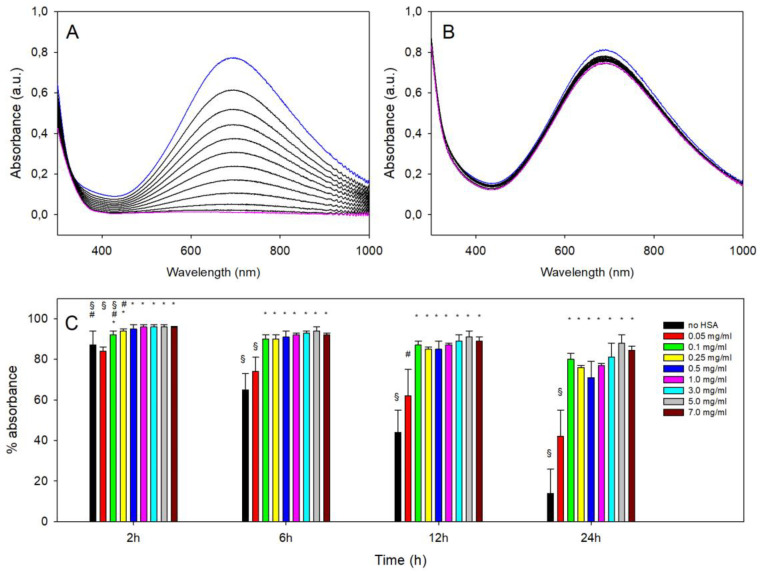
(**A**) Series of spectra recorded at 2 h intervals for citrate-coated PBnps at pH 7.4 (phosphate buffer); the spectrum at t = 0 and 24 h are in blue and pink color, respectively, while all the spectra at intermediate times are in black; (**B**) same, for PBnp@HSA prepared with C_HSA_ = 5 mg/mL; (**C**) percentage of residual absorbance after 2, 6, 12, and 24 h for untreated (citrate-coated) PBnps and for PBnp@HSA prepared with all C_HSA_ concentrations; different symbols correspond to the classification groups within the same analyzed time (i.e., 2 h, 6 h, 12 h, or 24 h) for the ANOVA Tukey’s test (*p* < 0.05).

As for all Fe^3+^ aqueous complexes, the coordinated water molecules can undergo hydrolysis to a hydroxide complex. This modifies the energy states involved in the charge transfer transition in the Fe^2+^···CN···Fe^3+^ system, resulting in significant spectral changes. We carried out a pH/spectrophotometric titration using PBnp@HSA prepared with C_HSA_ 1 mg/mL. The addition of 100 μL HCl 0.05 M to 24 mL PBnp@HSA shifted the pH to 3.51, with no significant changes in the absorption band position and intensity. Back titration with microaddition of 0.05 M NaOH was carried out until pH 9, obtaining the series of spectra displayed in Figure 2B. The titration was completed within 1.5 h to avoid the superimposition of significant spectral changes due to PBnp degradation (absorbance decreases of <4% after 2 h, for C_HSA_ 1.0 mg/mL, see Figure 3C. An equilibrium process is clearly observed, with an isosbestic point at 605 nm. The absorbance decreases, and the band blue-shifts with increasing pH. To evidence the trend of the titration in Figure 2B, the starting spectrum (pH 3.51) is red, and the last one (pH 9.03) is blue. The only anomalous absorption spectrum was obtained at pH 4.81 (green in Figure 2B), as it sits outside of the series and does not cross the isosbestic point, with a higher absorbance at all wavelengths, indicating increased scattering. A deeper insight into the observed process is obtained with Figure 2C, which displays the absorbance vs. pH at a representative wavelength (780 nm), blue triangles, and the zeta-potential vs. pH values measured for the same solution, black circles. The zeta-potential is positive in a strongly acidic solution. It goes near 0 mV (+2.6 mV) at pH 4.81 and becomes negative at higher pH. This is due to the charge of the HSA corona, in agreement with the HSA isoelectric point (pI) of ~5.0 [18]. The zeta-potential vs. pH points can be fitted with a sigmoid (Figure 2C, black dashed line), providing a calculated inflection point at pH 5.18, well in agreement with the reported HSA pI. When the zeta-potential is ~0, the PBnp@HSA agglomerate, with increased scattering in the absorption spectrum (green spectrum in Figure 2B). The positive and negative HSA charge at lower and higher pH, respectively, instead prevents agglomeration by electrostatic repulsion. DLS measurements carried out during the titration (SI4) confirm this picture, with d_h_ values in the 140–150 nm range for all the pH values except for the 4.81 point (d_h_ = 880 nm). Also, the Abs_780_ vs. pH points follow a typical weak acid titration profile that can be fitted with a sigmoid (blue dashed line in Figure 2C). However, in this case, the inflection point is at pH 6.68, corresponding to the pKa of the water molecules coordinated to surface Fe^3+^ ions in PBnp@HSA. While the Fe^3+^ aquaion [Fe(H_2_O)_6_]^3+^ has a much lower pKa value (2.73) for the first water deprotonation [19], a 6.68 pKa value is common for a Fe^3+^ cation bound to five ligands and with the sixth coordination position occupied by a water molecule (e.g., pKa = 7.16 in a Fe^3+^ complex with a pentadentated salen-like ligand [20]). Noticeably, a pH-spectrophotometric back titration with strong acid on the basic PBnp@HSA solution displays the reverse behavior, with absorbance increase and red-shift of the CT band (SI5), while keeping the isosbestic point at pH 6.05, as expected for a reversible equilibrium.

### 3.3. Stability vs. Decomposition of PBnp@HSA at pH 7.4 as a Function of C_HSA_

The stability of PBnp@HSA at physiological pH was studied by adding 1 mL of H_2_PO_4_^−^/HPO_4_^2−^ buffer at pH 7.4 to 2 mL of a PBnp@HSA solution, prepared as described in Section 2.3 and diluted with water (before buffer) to obtain an absorbance value of ~1.5 on the absorption maximum. An absorption spectrum was recorded immediately after mixing and every 2 h until 24 h. This was performed for all the PBnp@HSA prepared with the examined set of C_HSA_, ranging from 0.025 mg/mL to 7.0 mg/mL. For the sake of comparison, a solution of citrate-coated PBnps was also treated with the phosphate buffer at pH 7.4. In this latter case, a dramatic decrease in the charge transfer band was observed, as we have already reported [8], and as is shown in Figure 3A. When HSA was used to coat PBnps, we noticed a stabilization at any C_HSA_ concentration. Figure 3B displays the representative series of spectra measured at pH 7.4 on a PBnp@HSA prepared with C_HSA_ 5.0 mg/mL, in which minor spectral variations are observed in a 24h interval. The treatment with 7.4 phosphate buffer was repeated 3 times for PBnp@HSA prepared with all the C_HSA_ concentrations. Absorbance data at 690 nm were extracted at 2, 6, 12, and 24 h and compared with the starting value to calculate the % of residual absorbance. The averages of the three repetitions are shown in Figure 3C and compared with the % of residual absorbance observed with no HSA corona (black bars).

While PBnp@HSA prepared with C_HSA_ ≥ 0.5 mg mL maintain their spectral features nearly intact at 12 h (and also at 24 h for C_HSA_ = 3 mg/mL or higher, with residual % absorbance > 80%), decomposition is evident even after 6 h with lower C_HSA_ values. Even if the PBnp@HSA prepared with low C_HSA_ are significantly more resistant than uncoated PBnps at pH 7.4, these synthetic conditions are insufficient to impart proper protection. Moreover, after the treatment of PBnps with low C_HSA_, ultracentrifugation provided not a pellet but a strip of blue material stuck to the tube wall (photographs in SI6) that was hard to redissolve, needing a long ultrasound treatment. In addition, the redissolved solutions, either as such or treated with the phosphate buffer, could randomly give aggregates and precipitation (SI6) after 2–3 h. The tendency toward aggregation is also confirmed by the d_h_ data listed in Table 2, showing large dimensions (>200 nm) and higher PDI for preparations carried out with low C_HSA_.

The fact that a small absorbance decrease (12–19%) was observed after 24 h for PBnp@HSA prepared even with the highest C_HSA_ (3–7 mg/mL range) prompted us to simulate the injection of citrate-coated PBnps in an environment with HSA concentration comparable to that of the blood serum. A 200 μL portion of a ~10× concentrated PBnp solution was treated with 100 μL of a pH 7.4 phosphate buffer that also contained 120 mg/mL HSA (details in Section 2.6) to obtain a solution of PBnps with 40 mg/mL HSA. This mimics the albumin concentration in human blood serum (35–50 mg/mL [11]). Using a 1 mm cuvette, spectra were recorded every 2 h until 24 h, observing a very similar behavior as for C_HSA_ 3–7 mg/mL (see SI7). In particular, after 24 h, we observed a 18% decrease in the band. Noticeably, in the serum-like conditions experiment, the PBnps bear both the hard and soft corona, while in the standard preparative conditions, only the hard corona is maintained following the ultracentrifugation/redissolution step after equilibration with HSA. This suggests the preponderant role of the HSA hard protein corona in stabilizing PBnps against decomposition in in vivo-like conditions. Moreover, the observation that after 24h in serum-like conditions, 82% of the PBnps were still present suggests taking integrity > 80% after 24 h to evaluate the effectiveness of HSA protection in PBnp@HSA, and this is obtained when preparations are carried out with C_HSA_ 3.0 mg/mL or higher (Figure 3C).

### 3.4. Quantitative Determination of HSA Bound to PBnps

The quantitative determination of the HSA protein corona has been carried out by modifying the Lowry method to suit our conditions. The Lowry method is a spectrophotometric protocol carried out in aqueous solutions that uses the Folin–Ciocalteu reagent for the quantitative determination of proteins [12]. In this method, a basic Cu(II) solution interacts with proteins in the presence of the tartrate anion, forming a Cu(II) complex with four peptide nitrogen atoms, similar to the biuret method [21]. Moreover, the Folin–Ciocalteu reagent is added, which contains phosphomolybdate and phosphotungstate anions that are reported to interact with the complexed Cu(II) ions, generating a large absorption band in the 650–750 nm range. Although the complete sequence of reactions remains unclear, this is an established colorimetric protocol that allows the detection of total proteins in water in the 5–100 μg/mL range [21]. However, the Lowry method may have a number of interfering species [21], and we must consider that PBnps also absorb in the 600–750 nm range.

**Figure 4:**
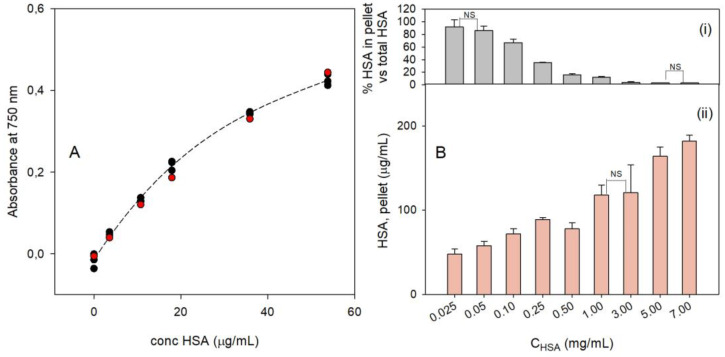
(**A**) Calibration points (black circles) and fitting curve (dashed curve) using the Lowry method with different concentrations of HSA and PBnp solutions as the background; the red circles are points obtained using bidistilled water as the background; (**B**) (**i**) % HSA bound to PBnp (p-C_HSA_) vs. total HSA added in the synthesis (C_HSA_); (**ii**) concentration (μg/mL) of HSA bound to the pellet vs. total HSA added in the synthesis (C_HSA_); data in panel (**i**,**ii**) are significantly different at the ANOVA Tukey’s test (*p* < 0.05) except the couples marked with NS.

Due to this, we recorded a calibration curve (details in Section 2.8.1) using a standard HSA solution and by adding measured quantities of HSA to a PBnp solution (identical to that used to prepare PBnp@HSA) instead of pure water. In agreement with the Lowry method, absorbance values were collected at 750 nm. The calibration points were recorded in three different runs with HSA concentrations in the 0–110 mg/mL range. At HSA concentrations higher than ~60 μg/mL, the observed Abs_750_ values from the three runs were significantly scattered, while their average values became almost identical (see SI2). Due to this, we considered only the 0–60 μg/mL HSA concentration range, where the collected Abs_750_ values are almost superimposable at a chosen HSA concentration (Figure 4A). The points were fitted with a cubic function (Figure 4A, dashed curve) that was used to determine experimental HSA concentrations for the prepared PBnp@HSA solutions. For the sake of comparison, we also collected Abs_750_ at the same HSA concentrations of the calibration curve but using pure water instead of a PBnp solution. The obtained points (red circles, Figure 4A) are very similar to those obtained with PBnps, indicating a small if not negligible interference of the PB color.

The HSA concentration was determined for all the PBnp@HSA solutions prepared with all the different C_HSA_ used in their synthesis (0.025–7.0 mg/mL), always performing measured dilutions wherever the expected maximum concentration would exceed the 0–60 μg/mL range. The PBnp@HSA solutions were prepared as described in Section 2.3. After the addition of HSA and 2 h equilibration time, 10 mL PBnp@HSA samples were ultracentrifugated, and the supernatant (9.5 mL) was separated from the pellet, leaving 0.5 mL supernatant over the latter to prevent accidental removal of part of it. Then, 9.5 mL bidistilled water was added to the pellet, reintegrating the starting 10 mL volume. The HSA concentration was determined on the supernatant (s-C_HSA_) and on the redissolved pellet (p-C_HSA_). In the latter case, we subtracted the contribution to HSA concentration due to the residual 0.5 mL of supernatant. The results are listed in Table 3.

Starting from C_HSA_ 0.100 mg/mL to the highest concentration used in the preparation of PBnp@HSA (C_HSA_ 7.0 mg/mL), the sum of p-C_HSA_ and s-C_HSA_, i.e., the total experimental C_HSA_, is very close to the calculated, expected one. This is shown by the last column of Table 3, which reports %_HSA_E/C, i.e., the percent of the total experimental C_HSA_ vs. calculated C_HSA_, with %_HSA_E/C = 100 × (p-C_HSA_ + s-C_HSA_)/C_HSA_. The values of %_HSA_E/C are ~100% in the mentioned range. Only for the two lowest C_HSA,_ the %_HSA_E/C value largely exceeds 100, most probably due to scattering, i.e., to the formation of turbidity due to the already observed tendency to aggregate of PBnp@HSA prepared with low C_HSA_. Figure 4B, panel (ii) visually displays p-HSA vs. C_HSA_, showing an almost linear increase in the HSA bound to PBnps with the increase in C_HSA_. Moreover, in all the cases for which %_HSA_E/C does not exceed 100%, the number of HSA molecules per PBnp can be safely calculated as the ratio between the protein bound to PBnp (p-C_HSA_) and the concentration of the nanoparticles (1.17 × 10^−9^ M). We found numbers much higher than the 180 units calculated in Section 3.1, ranging from ~1000 HSA/PBnp for C_HSA_ 0.250 mg/mL to ~1500 HSA for C_HSA_ 3.0 mg/mL and ~2300 HSA/PBnp for C_HSA_ 7.0 mg/mL. This indicates that the formation of the “hard” HSA corona is an equilibrium process, pushed toward the product by the mass effect, and that the “hard” corona is not just a monolayer on the PBnp surface but indeed a thicker, multilayered coating. Panel (i) in Figure 4B shows the % HSA bound to PBnps in the pellet vs. C_HSA_. While for low C_HSA_ values, the proteins forming the corona are all the added or the large majority of it (coherently with the very low s-C_HSA_ values; Table 3), at high C_HSA,_ only a small fraction (3–4%) adheres to the PBnp surface. These observations fit with the stability data observed at pH 7.4 (Figure 3C and with the d_h_ data (Table 2): at C_HSA_ 0.025–0.100 mg/mL, the quantity of HSA adsorbed on the PBnp@HSA surface is not sufficient to give a protein corona capable of protecting the coated particles from basic degradation. Moreover, insufficient coating promotes PBnp aggregation even at pH 7.4, a phenomenon that could be driven by bridging HSA between partially coated PBnp@HSA.

## 4. Conclusions

While PBnps have become extremely popular in nanomedicine studies [1,4,5], to the best of our knowledge, this is the first study on the formation of a protein corona around them. Differently from most studies of the protein corona on different types of nanoparticles [17], in this paper we focused our attention on phenomenological aspects, in particular on the stability gained by PBnps thanks to the formation of an HSA corona. This aspect is particularly relevant for in vivo and in vivo-like studies on PBnps, as HSA is the most abundant blood protein, and we have previously demonstrated that in a solution at the pH of blood, i.e., 7.4, PBnps are intrinsically unstable and decompose in a few hours. In this context, we have shown that the formation of an HSA corona around PBnps is actually capable of stabilizing the latter from basic degradation at pH 7.4. We explored the quantitative aspects of an HSA corona formation, as we believe that these may be particularly useful for the large bunch of scientists working with PBnps in the nanomedicine context. In particular, we found that when the PBnps prepared in this paper were treated with C_HSA_ ≥ 3.0 mg/mL, more than 80% of the PBnp@HSA were intact after 24 h in a buffer at pH 7.4, a value comparable to what was obtained when the actual HSA concentration in blood was tested. Quantitative determination of the HSA bound to PBnps also allowed us to determine that the number of HSA molecules adhering to PBnps largely exceeds the 180 HSA units per PBnp calculated with simple space-filling considerations, pointing toward a multilayered protein hard corona. In particular, taking C_HSA_ 3.0 mg/mL as the lowest suitable value to obtain PBnp@HSA with a robust stability at pH 7.4, it can be calculated that a minimum value of 1500 HSA molecules adhering to a single cubic PBnp of 41 nm side is required. The corresponding average number of HSA molecules per surface unit (nm^2^) is thus 0.15 HSA/nm^2^. This result is of general utility when stable HSA-coated PBnps of any dimension are to be prepared for use in in vivo-like conditions. Moreover, the stabilization brought by the protein corona also allowed for the first time the pH-spectrophotometric study and rationalization of the deprotonation of the H_2_O molecules bound to the surface Fe^3+^ ions of PBnps and of its effect on the PBnp optical properties. These have a pKa of 6.68 and are responsible for the PBnp λ_max_ shift from 706 nm (acidic solution) to 685 nm (basic solution). As a final remark, it is interesting to note that the latter study also revealed that pH influences the charge of the HSA corona, leading to aggregation at pH~5 (zeta-potential of PBnp@HSA~0), a value corresponding to HSA isoelectric point. Such a behavior should be considered in cases in which PBnp@HSA could be uptaken by cells and, in particular, following the typical endocytosis pattern, as in endosomes the pH is ~5 [22].

## Figures and Tables

**Table 1:** Composition and concentration of the standard solutions used to obtain the Abs750 vs. C_HSA_ calibration curve.

	Solution E (mL)	Bidistilled Water (mL)	Solution D (mL)	1 N Folin Reagent (mL)	HSA Concentration (μg/mL)
blank	0.00	5.00	0.70	0.10	0
Std 1	0.10	4.90	0.70	0.10	3.59
Std 2	0.30	4.70	0.70	0.10	10.76
Std 3	0.50	4.50	0.70	0.10	17.93
Std 4	1.00	4.00	0.70	0.10	35.86
Std 5	1.50	3.50	0.70	0.10	53.79
Std 6	2.00	3.00	0.70	0.10	71.72
Std 7	2.50	2.50	0.70	0.10	89.65
Std 8	3.00	2.00	0.70	0.10	107.59

**Table 2:** Data for PBnp@HSA ^1^.

C_HSA_ (mg/mL)	d_h_ (nm)	PDI ^2^
0.025	320(8)	0.37(0.01)
0.050	291(3)	0.23(0.03)
0.10	240(1)	0.21(0.04)
0.25	183(1)	0.18(0.02)
0.50	176(2)	0.17(0.02)
1.0	142(1)	0.19(0.02)
3.0	123(2)	0.22(0.01)
5.0	112(2)	0.20(0.01)
7.0	113(2)	0.21(0.02)

^1^ Data obtained from 3 measurements, standard deviation in parentheses; ^2^ polydispersity index, standard deviation in parentheses.

**Table 3:** Quantitative data on HSA.

C_HSA_ (μg/mL)	p-C_HSA_^a^ (μg/mL)	s-C_HSA_^a^ (μg/mL)	% _HSA_E/C
25	48(6)	4.4(0.6)	210
50	58(5)	9(3)	134
100	72(6)	35(6)	107
250	89(2)	162(12)	100
500	78(7)	406(18)	97
1000	118(12)	899(103)	102
3000	121(33)	2566(101)	90
5000	164(11)	4574(471)	95
7000	182(7)	6627(390)	97

^a^ Average of three determinations, standard deviation in parentheses.

## Data Availability

Additional data are available in the Supporting Information.

## Supplementary Materials

The following supporting information can be downloaded at https://www.mdpi.com/article/10.3390/nano14161336/s1, SI1: Absorption spectra and collected data for the calibration curve, Lowry method; SI2: Same, in water; SI3: Additional TEM images; SI4: Hydrodynamic diameters of PBnp@HSA in the pH/spectrophotometric titrations; SI5: Absorption spectra in the back (basic to acid) pH/spectrophotometric titration; SI6: Photographs of ultracentrifugation test tubes for preparations with low C_HSA_; SI7: Absorption spectra vs. time for PBnps in 7.4 buffer with HSA in serum-like concentration.
