# Supplementary material for: Human Serum Albumin Protein Corona in Prussian Blue Nanoparticles
Source: Nanomaterials (Basel). 2024 Aug 11;14(16):1336. doi: 10.3390/nano14161336 (PMC11356891; doi:10.3390/nano14161336)
Supplement: Supplementary file 1 [file nanomaterials-14-01336-s001.zip › nanomaterials-3139218-supplementary.pdf]

nanomaterials

Human Serum Albumin Protein Corona in Prussian Blue Nanoparticles

Supplementary Information

SI1 – Absorption spectra and collected data for the calibration curve in the quantitative determination of HSA with Lowry method, Folin reagent.

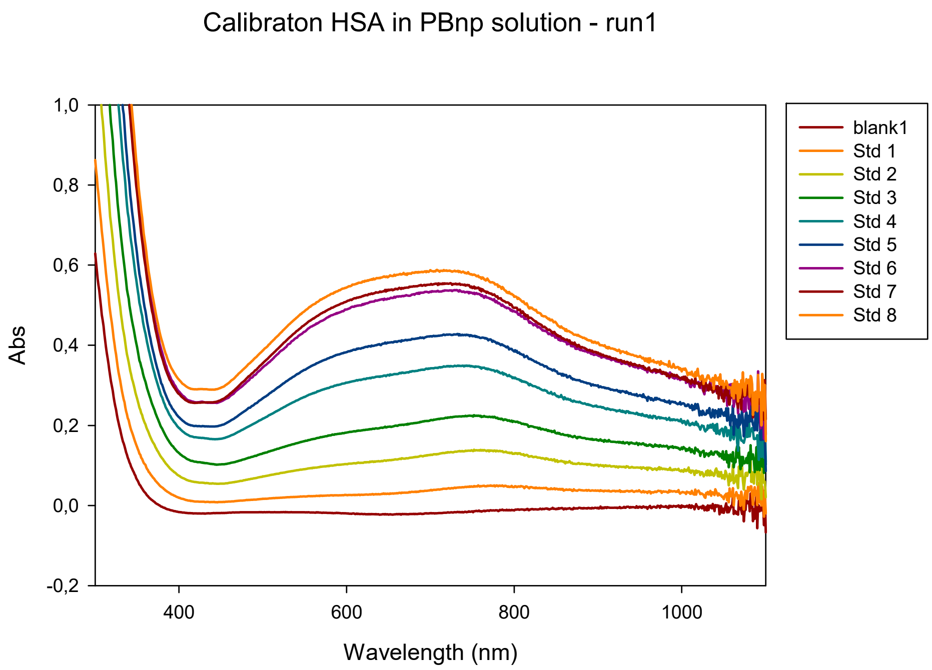

Collected data, RUN 1. Grey filled lines were not used for the calibration curve

**Run 1**		
	Nominal conc HSA in PBnp solution (µg/ml)	Abs 750 nm
blank	0	-0.0144
std 1	3.5862	0.047
std 2	10.7586	0.1373
std 3	17.931	0.2239
std 4	35.8621	0.3477
std 5	53.7931	0.4224
std 6	71.7241	0.5304
std 7	89.6552	0.5429
std 8	107.5862	0.5754

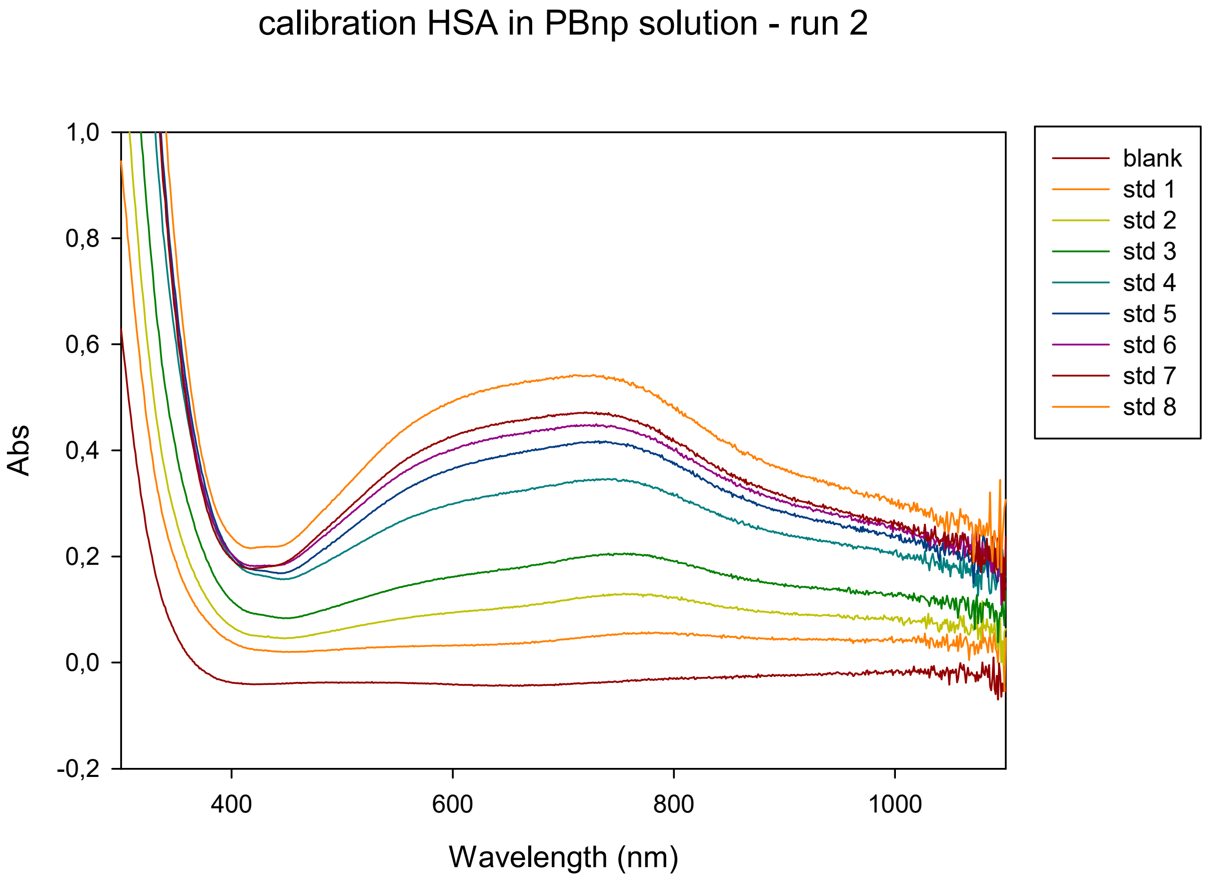

Collected data, RUN 2. Grey filled lines were not used for the calibration curve

**Run 2**		
	Conc HSA in PBnp (μg/ml)	Abs 750 nm
blank	0	-0.0362
std 1	3.5862	0.0536
std 2	10.7586	0.1278
std 3	17.931	0.2044
std 4	35.8621	0.3418
std 5	53.7931	0.4126
std 6	71.7241	0.4419
std 7	89.6552	0.4656
std 8	107.5862	0.534

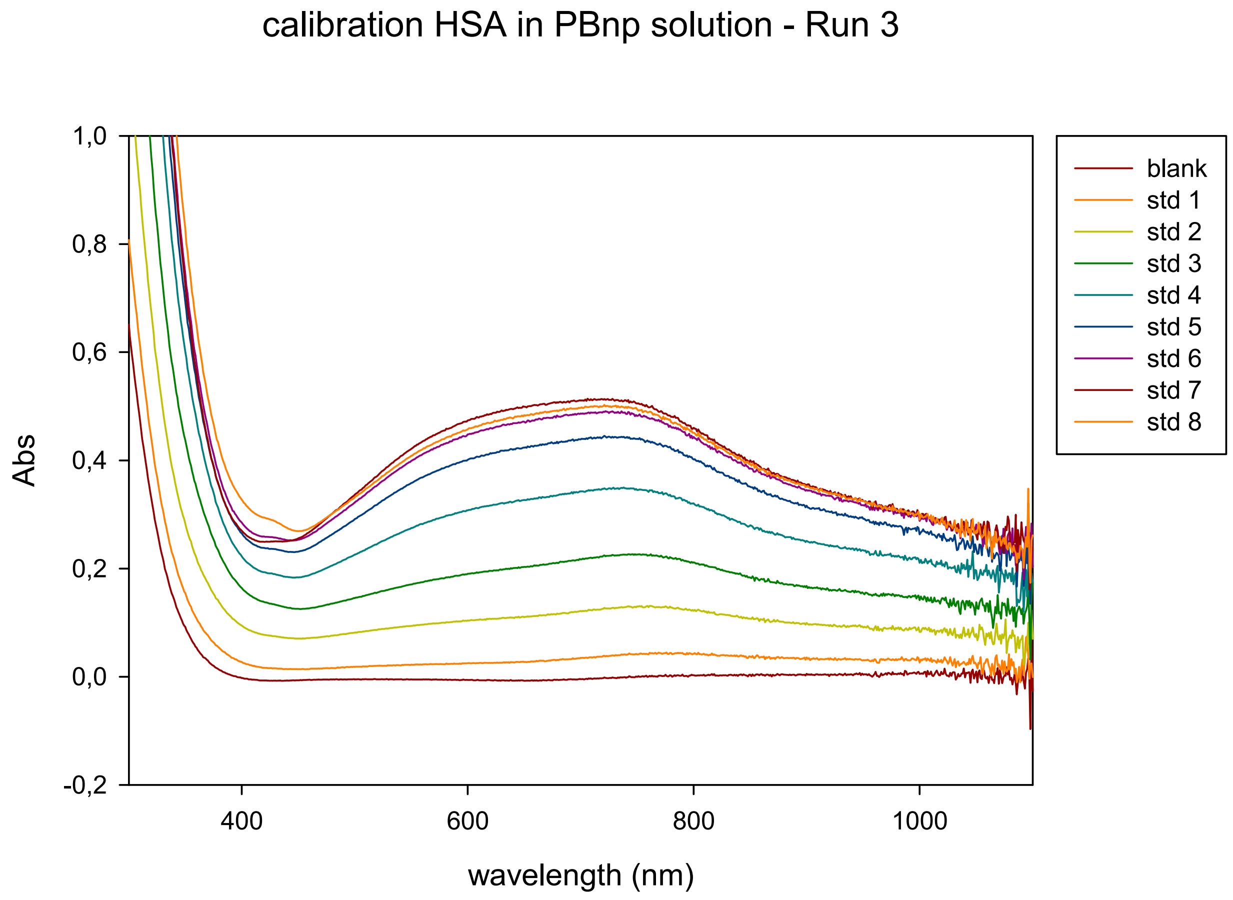

Collected data, RUN 3. Grey filled lines were not used for the calibration curve

**Run 3**		
	Conc HSA in PBnp (µg/ml)	Abs 750 nm
bianco	0	-4.23E-04
std 1	3.5862	0.0414
std 2	10.7586	0.1301
std 3	17.931	0.226
std 4	35.8621	0.3451
std 5	53.7931	0.4399
std 6	71.7241	0.4824
std 7	89.6552	0.5053
std 8	107.5862	0.4942

SI2 – Calibration HSA in water (no PBnp added)

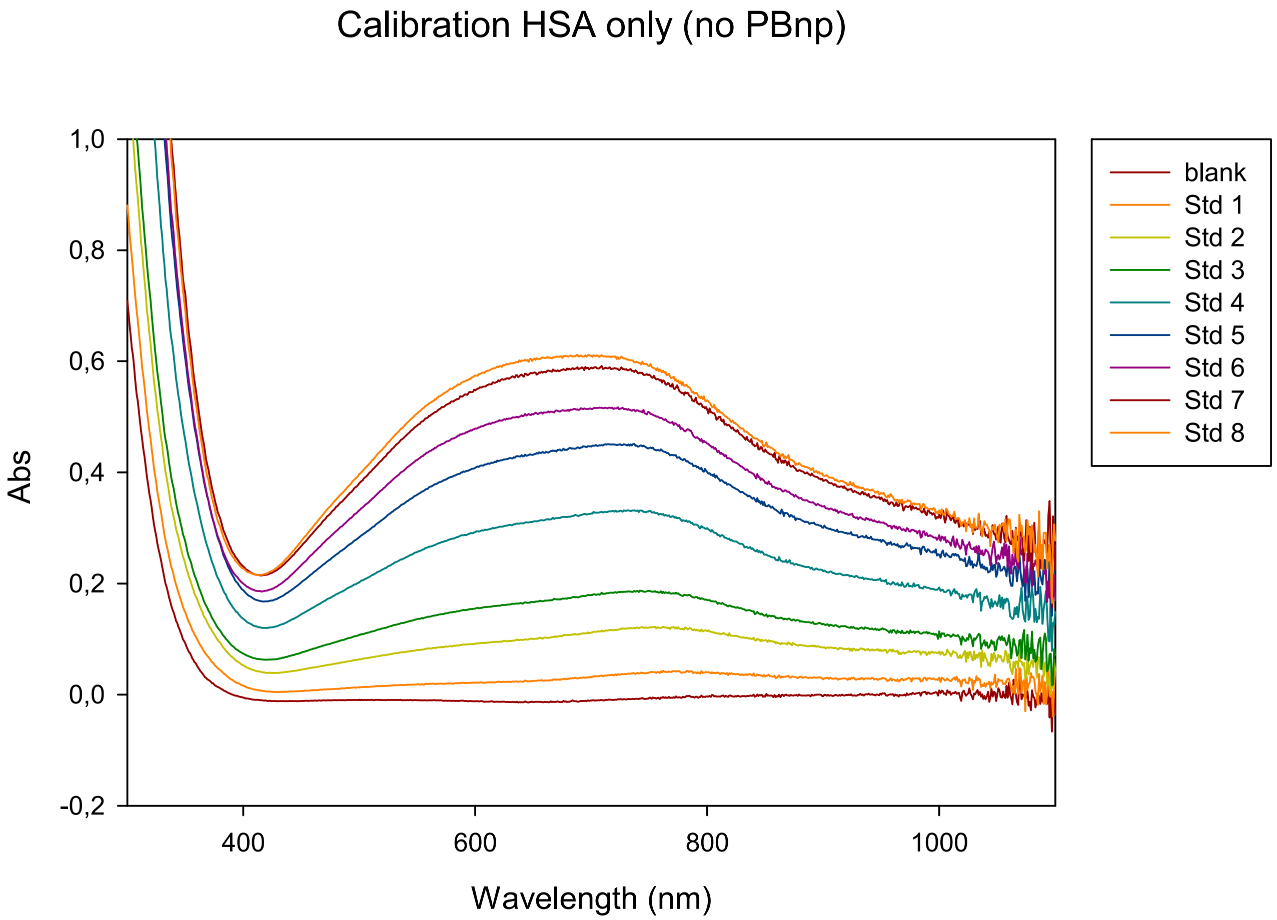

Collected data

	Conc HSA (μg/ml)	Abs 750 nm
blank	0	-5.55E-03
std 1	3.6207	0.0396
std 2	10.8621	0.1206
std 3	18.1034	0.1866
std 4	36.2069	0.3306
std 5	54.3103	0.4442
std 6	72.4138	0.5082
std 7	90.5172	0.5761
std 8	108.6207	0.5916

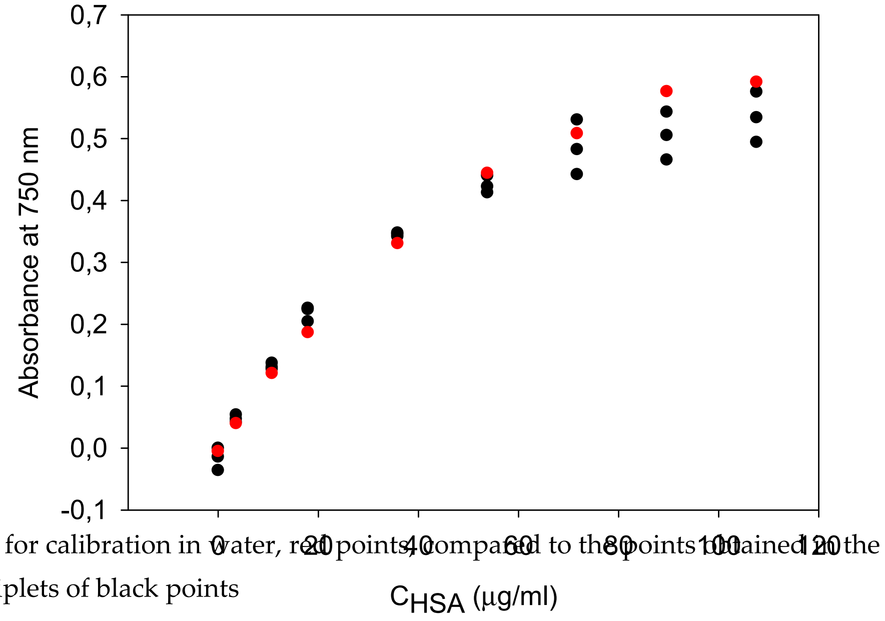

The graph shows the Abs750 vs C_{HSA} points for calibration in water, red points, compared to the points obtained in the previous section (HSA in PBnp solution), triplets of black points

SI3 – TEM images, PBnp@HSA prepared with C_{HSA} 5.0 mg/mL

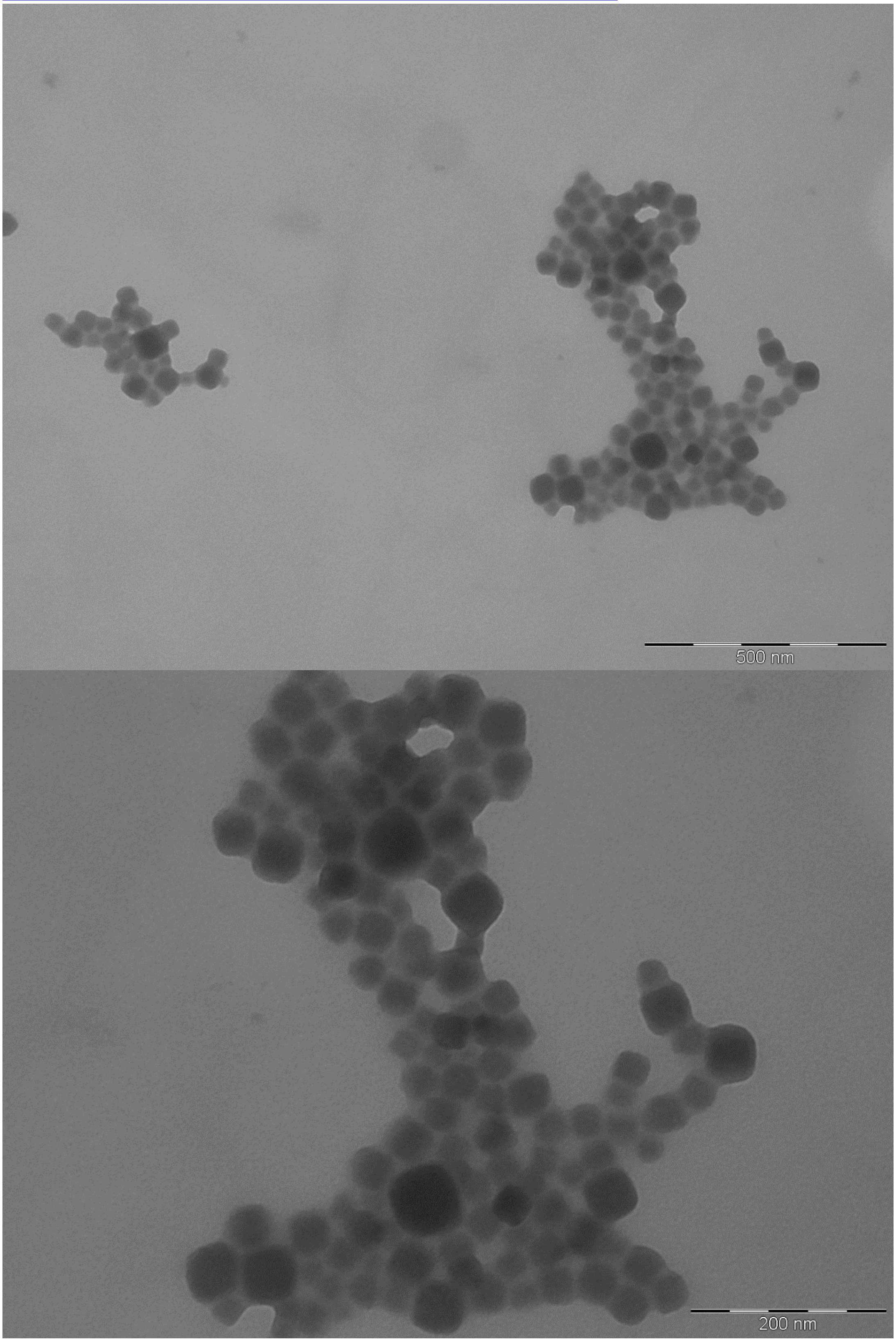

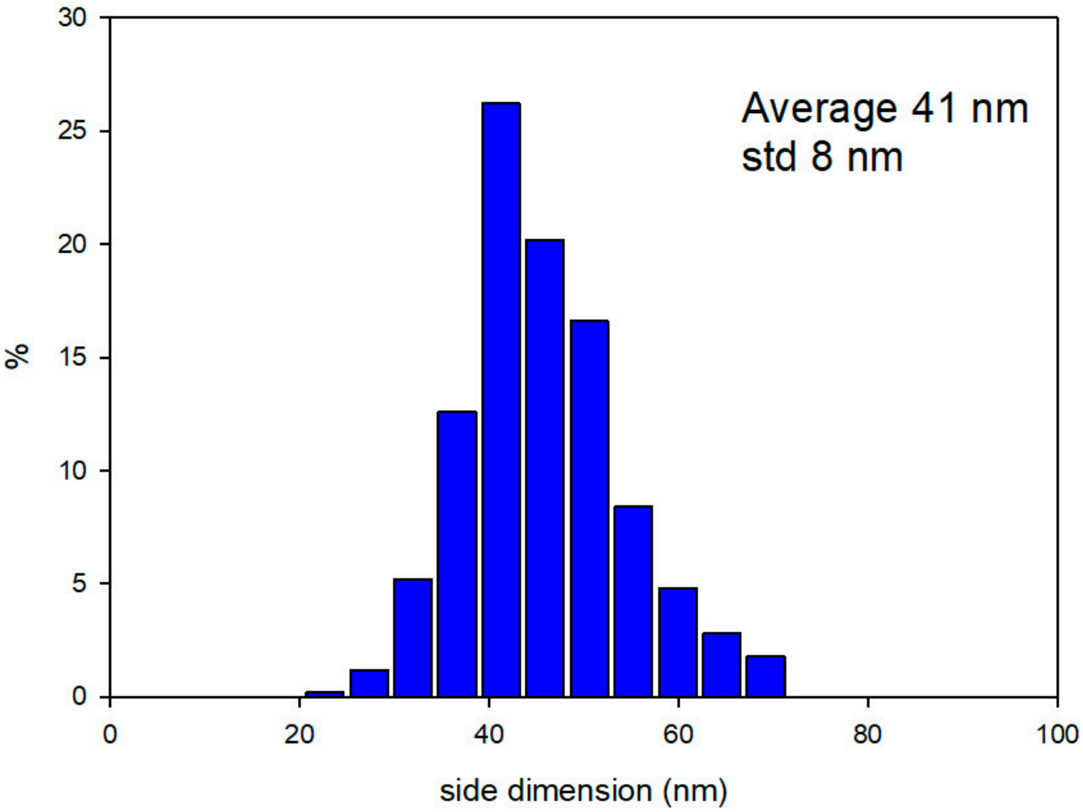

Histogram of PBnp dimensions, obtained from TEM images on citrate-coated PBnp

SI4: d_h values for HSA@PBnp (prepared with CHSA 1 mg/mL) during pH-spectrophotometric titration

pH	d_h (nm)
3,51	146
3.78	149
4.27	137
4.81	884
6.17	158
6.48	151
7.20	150
7.93	155
8.50	149
9.03	140

SI5 – absorption spectra for the back titration (from basic to acid) on HSA@PBnp

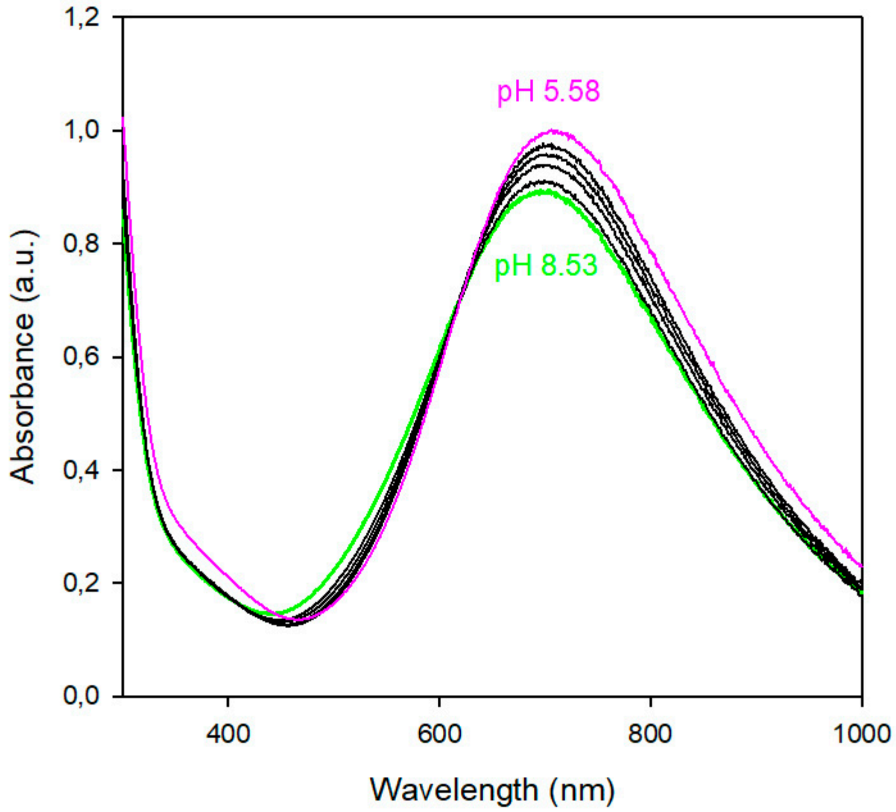

Figure SI5. The titration has been carried out by adding HCl (0.05 M) to the solution at pH 9.03 obtained at the end of the pH-spectrophotometric titration shown in the main text (Figure 2B). Here the absorbance increase and the band maximum shifts to the red. The first spectrum (pH 8.53) is evidenced in green, the last one (pH 5.58) in purple. Isosbestic point is at 605 nm

SI6: photographs of HSA@PBnp after ultracentrifugation, for low C_{HSA} preparations

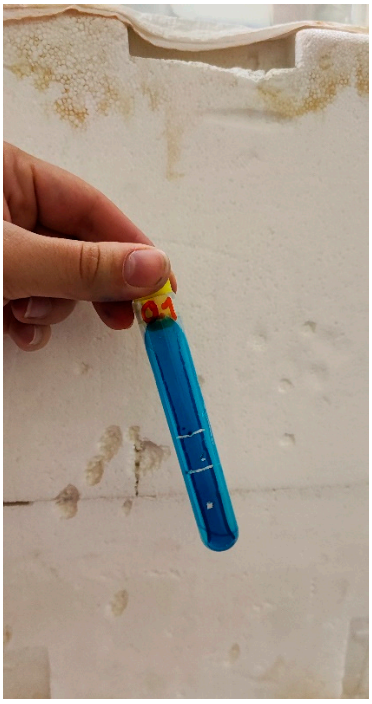

C_{HSA} 0.1 mg/mL

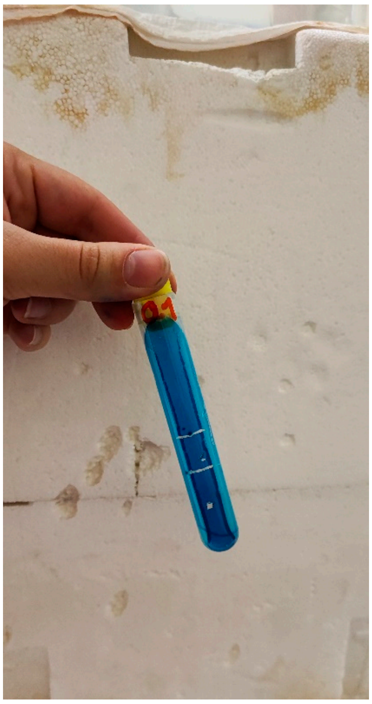

C_{HSA} 0.05 mg/mL

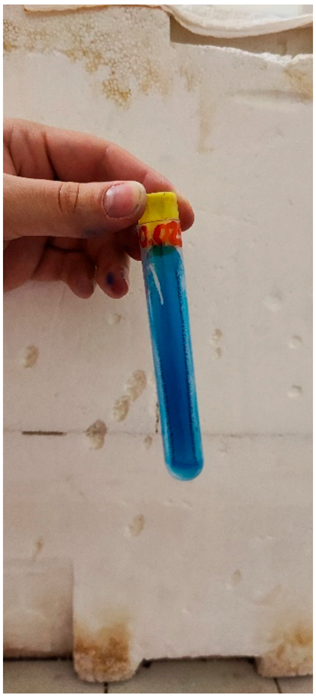

C_{HSA} 0.025 mg/mL

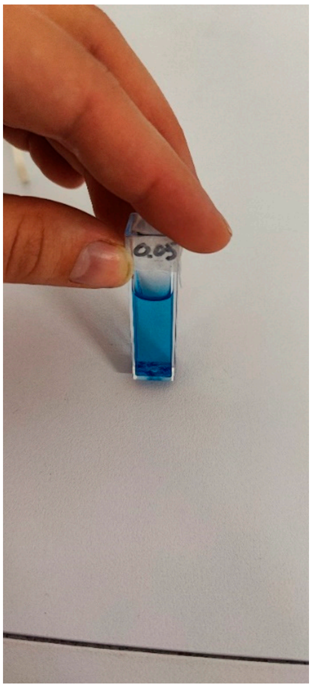

C_{HSA} 0.05 mg/mL, treatment of the redissolved solution with phosphate buffer at pH 7.4

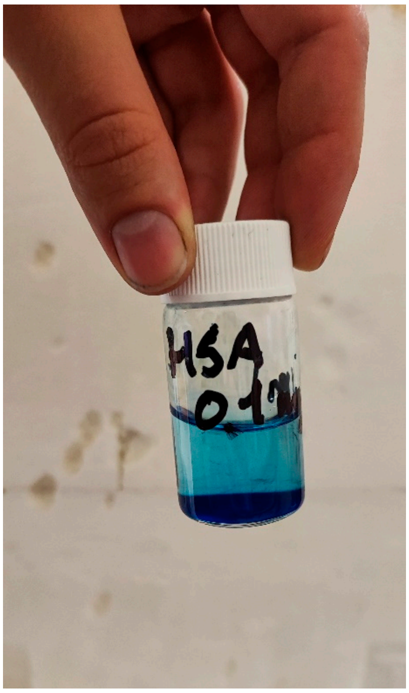

C_{HSA} 0.1 mg/mL, pellet redissolved in water, after 8 h

SI7: absorption spectra of PBnp treated with pH 7.4 phosphate buffer in serum-like conditions

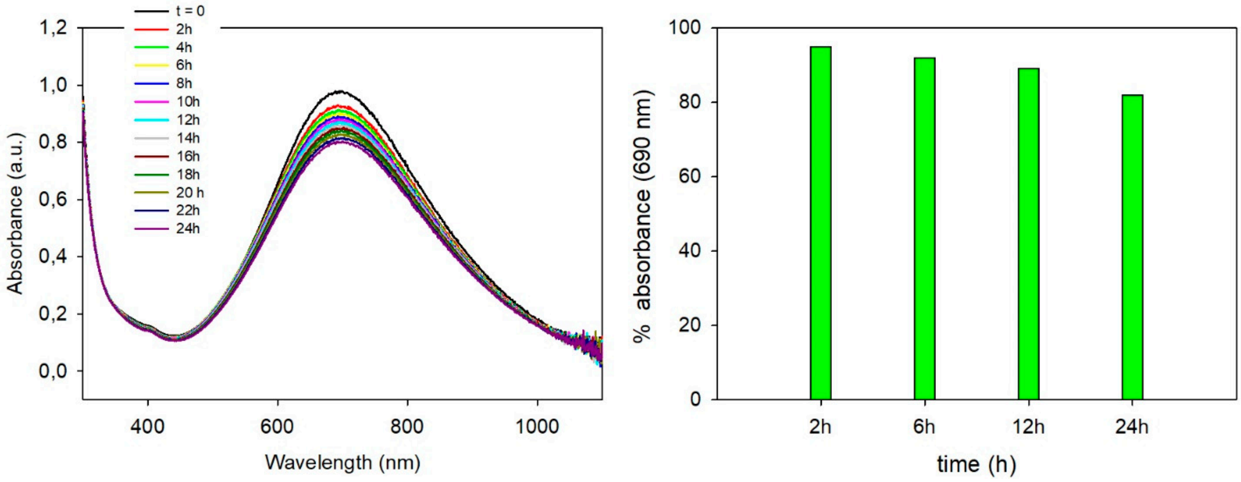

Left: series of spectra taken each 2 h until 24 h

Right: % of residual absorbance (690 nm) at 2, 6, 12, 24 h

The concentration of HSA is 40 mg/mL in the added buffer solution.
